# Quality of Life and Related Dimensions in Cancer Patients Treated with Mistletoe Extract *(Iscador)*: A Meta-Analysis

**DOI:** 10.1155/2012/219402

**Published:** 2011-06-14

**Authors:** Arndt Büssing, Christa Raak, Thomas Ostermann

**Affiliations:** Center for Integrative Medicine, Faculty of Health, University of Witten/Herdecke, Gerhard-Kienle-Weg 4, 58239 Herdecke, Germany

## Abstract

*Objectives*. The aim of this meta-analysis was to determine the effectiveness of the fermented plant extract *Iscador*, produced from the white-berry European mistletoe, in the treatment of patients with cancer with respect to quality-of-life- (QoL-) associated measures. *Methods*. We searched databases such as PubMed/Medline, Excerpta Medica Database (EMBASE), CAMbase, and other for controlled clinical studies on parameters associated with QoL. Outcome data were extracted and converted into standardized mean differences and their standard errors. *Results*. Thirteen prospective and controlled studies which met the inclusion/exclusion criteria reported positive effects in favor of the *Iscador* application. A random-effect meta-analysis estimated the overall treatment effect at standardized mean difference = 0.56 (CI: 0.41 to 0.71, *P* < .0001). However, the methodological quality of the studies was poor. *Conclusions*. The analyzed studies give some evidence that *Iscador* treatment might have beneficial short-time effects on QoL-associated dimensions and psychosomatic self-regulation.

## 1. Introduction

Complementary and alternative medicine (CAM) has become increasingly popular over the last decades. According to Bausell et al., especially patients with chronic diseases increasingly seek for CAM-therapies [1]. With a growing amount of health information in the internet, physicians and therapists and patients are often not prepared to judge provided information of CAM health care approaches properly. Information dissemination of published evidence about the effectiveness of remedies and therapies therefore forms a necessary basis for shared decision making for patients and practitioners. 

In Europe, extracts from *Viscum album* (VA-E), the European white-berry mistletoe, are widely used to treat patients with cancer, in addition to patients with arthrosis, hypertension, arteriosclerosis, diabetes, and so forth [2] Historically, the intentions of mistletoe uses were manifold and conflicting in several cases (i.e., swellings or tumors, epilepsy, diseases of spleen and liver, labor pains, “weakness of the heart,” edema, eczema, ulcers of the feet, burns, and granulating wounds) [2]. In 1920, mistletoe extracts were introduced for the first time as a cancer treatment by Steiner (1861–1925) [3], the founder of anthroposophy. He recommended a drug extract produced in a complicated manufacturing process combining sap from mistletoe harvested in the winter and summer [4]. Based on his recommendations, several anthroposophical doctors have treated their cancer patients with these extracts within the last century. 

Meanwhile, clinical evaluations of mistletoe as an adjuvant cancer treatment have expanded. Recent scientific research has confirmed that mistletoe extracts induce apoptosis, stimulate immunocompetent cells, and protect the DNA of mononuclear cells (for review see [2, 5, 6]). Several experiments using tumor-bearing animals showed impressive reduction of tumor growth and/or increased survival with the application of mistletoe therapy (for review see [2, 5, 6]). There are several whole-plant extracts from *Viscum album* on the market which differ with respect to the extraction process and thus relative proportions of their constituents [7]. Due to this diversity of mistletoe products and their proportions of pharmacologically relevant constituents, the interpretation of clinical studies is difficult. Consequently it is not too surprising that preceding reviews on the clinical effects of mistletoe extracts in cancer patients, which summarizes a mixture of studies with different designs and plant extracts used, are conflicting in their results [6, 8–13]. Several of these reviews detected heterogeneity of studies with respect to the drug extracts used to treat the patients and thus are not suited to calculate reliable effects sizes. In fact, there are up to five different pharmaceutical processes of mistletoe extract preparation, and thus the pharmaceutical products show highly specific pattern of active components (i.e., pattern of cytotoxic mistletoe lectins, viscotoxins, etc.) [7]. To overcome this problem, we intended to determine the effectiveness of VA-E in the treatment of patients with cancer and focused on the most commonly used VA-E which is covered by a relatively large spectrum of published studies, the fermented plant extract *Iscador *(WELEDA AG, Switzerland). This whole-plant extract is produced from fresh leafy shoots and fruits of the summer and winter harvest, is rich on mistletoe lectins and viscotoxins [7, 14], and is recommended to be applied 2-3 times per week subcutaneously.

While most clinical studies on the effects of VA-E focus on the survival of cancer patients, the effects on the patients' quality of life (QoL), which gains more and more importance as a relevant outcome variable in cancer therapy, received less consideration so far. Thus, we determined the effectiveness of the VA-E *Iscador* in the treatment of patients with cancer with respect to QoL-associated dimensions and analyzed the studies with respect to trials where patients of the control group received only standard care and no extra treatment.

## 2. Materials and Methods

### 2.1. Search Strategy

Between February and April 2008, we searched databases such as PubMed/Medline, the Excerpta Medica Database (EMBASE), the Cochrane Library, database of DIMDI (Deutsches Institut für Medizinische Dokumentation und Information), and CAMbase for clinical studies focusing on QoL-associated measures of cancer patients using *Iscador *extracts. Search terms were either “*Iscador*” and “study”, “mistletoe” and “study”, and “Viscum” and “study”. Finally we asked several experts for gray literature not listed in the above-mentioned databases, checked the reference lists of relevant articles and authors, and contacted the manufacturer of the VA-E. We performed an additional check for new studies in 2010, which did not reveal new results.

### 2.2. Selection Criteria

Inclusion criteria were all controlled clinical studies (at least historic or literature) on parameters associated with quality of life in cancer patients treated with the VA-E *Iscador*, published in English or German language journals. Neither the experts in the field nor the manufacturer was aware of any other study published in French, Spanish, Chinese, or other languages. However, one study from Denmark was published in a Danish journal and in an English language journal, and thus we referred to both publications presenting data of the same study. We are not aware of unpublished studies on the effects of *Iscador *on QoL.

We excluded field reports, case series, case reports, studies without a control group, abstracts which proceeded a full-length publication, translations of already published manuscripts, double publication of similar data (with the exception of the presentation of further data), internal reports, and unpublished manuscripts.

### 2.3. Analysis of Data

Two review authors independently assessed trials for inclusion in the review. They took part in the extraction of data and assessment of methodological quality and external validity. Disagreements were resolved by consensus. We graded the methodological quality of the studies by the following checklist (rater assessment): adequate description of the design (retrospective, prospective, retrolective, multicenter study, etc.), accrual (randomization, matched pairs, etc.), comparability of groups (controls and VA-E), description of dropouts, allocation concealment (analysis of concealed treatment allocation was difficult because most studies did not provide sufficient data to judge—either there were no statements or information was at least unclear), description of the intervention (dosage and duration of VA-E application), description of statistical analysis, and external validity (representative patients, relevant therapeutic concepts, generalization of results). Additionally we referred to the JADAD rating score which assesses randomization, blinding, and dropouts [15].

The reporting of the results adhered to the MOOSE [16] and QUOROM [17] guidelines.

### 2.4. Statistical Analysis

If a trial was found to be eligible, assessments of its methodological quality were done independently by two reviewers (A. Büssing, T. Ostermann) and recorded on an especially predesigned data form together with the basic trial data and the extracted results. Allocation concealment was assessed in accordance with the Cochrane guidelines [18]:

  A = adequate (telephone randomization or using consecutively numbered, sealed, opaque envelopes);
  B = uncertainty about the concealment (method of concealment is not known);
C = inadequate (e.g., alternate days, odd/even date of birth, hospital number).

Disagreements on methodological quality ratings were discussed by both assessors until they reached a consensus.

Data were independently extracted by two persons (A. Büssing, T. Ostermann) and independently entered into a data form which was especially designed for trials on mistletoe by a third person (C. Raak). If the data entries differed, both reviewers were contacted to recheck the publications and were forced to come to a consensus, which could be reached in all cases.

Data on the following details were extracted:

- details of the publication (first author, country, year, journal),
- details on the dosage and application of *Iscador, *
- type, name, dosage and application of the control therapy/alternative therapies,
- grading and location of cancer,
- age and gender distribution of patients,
- methodological quality of the study (see above),
- outcome(s).

All data were separately analyzed for trials where patients of the control group received only standard care and no extra treatment (or placebo-controlled trials). QoL-associated outcome data (QoL questionnaires and scales: mean values and standard deviations, mean/median differences, effect estimates and confidence intervals, odds ratios, etc.) were extracted as they were given in the publication. They were converted into standardized mean differences (SMD) and their standard errors (STE) using standard formulas [18]. Effect sizes <0.5 indicate small effects and >0.8 large effects [19]. 

All studies were analysed in a single analysis, regardless of on what scale the results had originally been measured or reported.

The association between sample size and trial results was graphically displayed in funnel plots, by plotting effect sizes on the horizontal axis (in a logarithmic scale) against their standard errors, or against the total patient numbers, on the vertical axis [20]. Funnel plots are adequate instruments to detect small study size effects, including publication bias. In the absence of bias, results from small studies should scatter widely at the bottom of the graph, with the spread narrowing among larger studies. Publication bias (and also lack of equipoise) may lead to asymmetrical funnel plots. Moreover, the asymmetry of the funnel plot was further explored by a weighted linear regression analysis (metaregression) which modeled the log SMD as a function of its standard error [21]. Weights were chosen inversely to the squared standard error. From this model, the asymmetry coefficient (AC) was estimated as the slope of the regression line. 

Heterogeneity between trials was assessed by standard *χ*²-tests and the *I* ^2^ coefficient which measures the percentage of total variation across studies due to true heterogeneity rather than chance [22].

Overall estimates of the treatment effect were obtained from random-effect meta-analysis [23]. Additionally, from metaregression a predicted SMD was obtained for trials with a standard error as small as the smallest observed standard error of all included trials. The extent to which study-level variables were associated with SMDs was investigated by fitting multivariable metaregression models. The following variables were considered: standard error of SMD, tumor localization (breast, stomach, lung, colon, ovary, corpus, skin cancer yes/no), randomization (yes/no), and matched-pair comparison (yes/no)—due to the fact that all matched-pair studies were from the same source.

## 3. Results

### 3.1. Search Results

**Figure 1:**
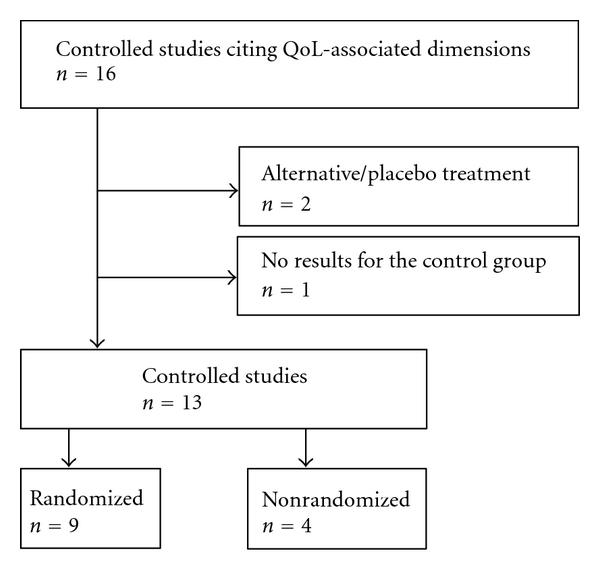
Flow diagram of study exclusion.

We found 16 studies on the clinical effects of *Iscador* usage on QoL-associated dimensions which were described in 11 publications (Table 1). Some described data on different sets of patients and/or tumor stages or different designs within the same report. Two randomized trials controlled *Iscador *against placebo/alternative treatment (i.e., water or vitamin B, resp. [24, 25]) and thus were not enrolled in the evaluation “*Iscador* versus no extra treatment.” The results of these studies are nevertheless presented in Table 1. Moreover, one study with a historic control, reporting just the QoL results of the VA-E arm [35], was excluded from the analysis. Thus, 13 studies provided data on QoL associated dimensions to extract SMDs and their standard deviations with respect to a comparison *Iscador *versus no extra treatment (Figure 1).

### 3.2. Design of Studies

All of the remaining 13 studies according to specifications in the articles had a prospective design. Nine of them were randomized (Figure 1). According to the nature of the control group, no trial was blinded. 

The oldest study (which was excluded from the analysis because of a lacking control group) dated back to 1984; all others were published in 2001 or later, the most recent being 2008. The number of patients enrolled varied considerably from 32 to 396; overall 734 patients were treated with *Iscador *and 741 patients served as controls. In most cases, the respective dosage of *Iscador*, was not given in the original studies.

The trials included in this meta-analysis were of poor quality, as indicated by randomization, matched-pair building, blinding, multicenter, description of dropouts, and so forth. (Table 1). Nine investigations reached a JADAD score of 2, five a score of 1, and one no point (Table 1). Due to methodological problems, an adequate blinding of VA-E application (which results in most cases in observable local reactions at the injection site) is not possible (reviewed in [10]), and thus the most important differentiating variable was in fact randomisation versus nonrandomisation and thus was used for the multivariable metaregression analysis.

### 3.3. Effect Sizes

**Figure 2:**
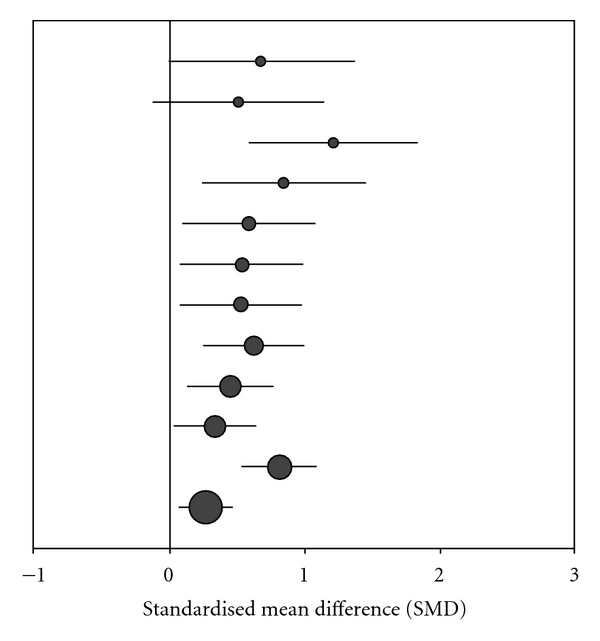
Standardized treatment effects (SMD and confidence intervals) of *Iscador* versus no extra treatment. The size of circles represents the weight of the study/strata in metaregression.

**Figure 3:**
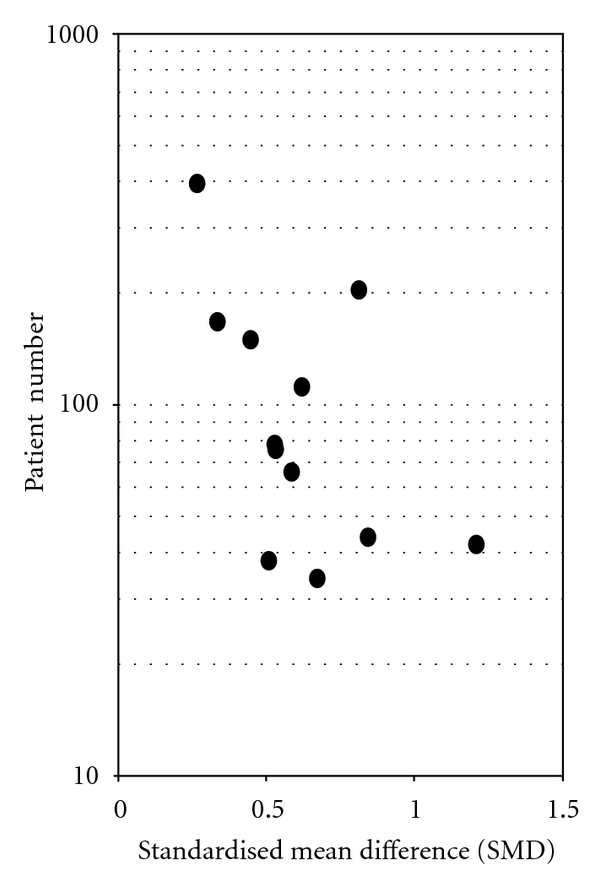
Funnel plots with respect to standard errors (the line in plot represents the regression line from metaregression): *Iscador* versus no extra treatment.

**Figure 4:**
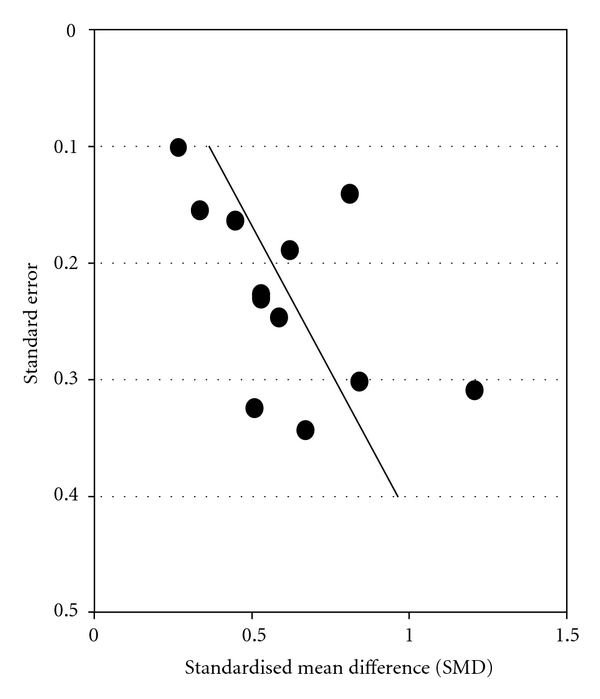
Funnel plots with total patient numbers (the line in plot represents the regression line from metaregression): *Iscador* versus no extra treatment.

As shown in Figure 2, all studies reported positive effects in favor of the *Iscador *application. Variability of study results was moderate (*I* ^2^ = 42.1%), but the funnel plot (Figures 3 and 4) showed considerable asymmetry with the largest investigation revealing the smallest effect (AC = 1.99, CI: 0.20 to 0.52, *P* < .0001). 

A random-effect meta-analysis estimated the overall treatment effect at SMD = 0.56 (CI: 0.41 to 0.71, *P* < .0001), indicating a moderate effect. In multivariable metaregression, neither tumor localization nor the design of the investigation turned out to be significantly associated with better or worse study outcome: breast cancer trials had a slightly better outcome than others (difference in SMD: 0.19, CI: −0.12 to 0.50, *P* = .22), randomized studies did not differ from nonrandomized (difference in SMD: −0.05, CI: −0.55 to 0.45, *P* = .84), and matched-pair studies (which were all using self regulation as a QoL-associated dimension) were comparable to others (difference in SMD: 0.01; CI: −0.55 to 0.45, *P* = .84).

## 4. Discussion

### 4.1. Quality of Studies and Outcome

Although the methodological quality of investigations on the clinical effects of VA-E has improved over the last years, as at least more randomized controlled studies were performed. However, many problems still remain: most trials did not report data on compliance and completeness of follow up, intention-to-treat analysis was rarely mentioned, and the number of patients was in all cases <200. Nevertheless, all studies were prospective and had a parallel group design; most were randomized, but none was blinded (Table 1). According to the JADAD score, the methodological quality of the enrolled investigations to assess the effects of VA-E on patients' QoL-associated variables is rather low (all studies <3). Randomisation versus nonrandomisation was thus the main relevant variable used in the multivariable metaregression analyses which showed that randomized studies did not differ from non-randomized investigations. 

In all publications addressing the effects of *Iscador* application on QoL-associated variables positive effects found; however, the funnel plots may indicate either a selection bias or a lack of equipoise (i.e., the investigators may have justifiable assumptions of drug superiority and thus intended to prove the effectiveness of the *Iscador*). Two-thirds of the studies were from the same origin and thus had the same methodological problems. These had a matched-pair design, either with or without randomization, a good description of the methodology, and a profound discussion of potential bias factors. It is obvious that the strict matching process significantly affected the number of patients enrolled in the evaluation (all studies had had sample sizes of <200 subjects), but it is difficult to explain the data of the funnel plots which indicate a publication bias in favor of positive results.

### 4.2. Congruence of Results

Although the benefit of adjuvant mistletoe treatment has been demonstrated in some randomized and observational studies, a comprehensive meta-analytical approach like the present one has not been previously conducted. Ernst et al. published a systematic review on randomized clinical trials (RCTs) using various VA-Es and stated that “statistical pooling was not possible because of the heterogeneity of the primary studies; therefore a narrative systematic review was conducted” [11]. We could confirm the heterogeneity of investigations on the clinical effects of VA-E, but nevertheless were able to extract data from several trials which provided enough data to calculate SMDs and their standard errors. Ernst et al. stated that the weaker studies implied benefits of VA-E, particularly in terms of quality of life, while none of the methodologically stronger studies were able to verify a benefit with respect to survival or QoL [11]. 

A Cochrane Review of Horneber et al. published in 2008 analyzed RCTs on various mistletoe extract preparations and indicated weak evidence that VA-E application could be effective with respect to QoL during chemotherapy for breast cancer [12]. Both groups [11, 12] argued that the main reason for the restricted informative value of the findings concerning subjective outcomes in trials with VA-E was the unblinded assessments or the unblinding of the intervention through local reactions. 

Also Kienle et al. summarized in their systematic review of various RCTs from 2003 that most of these publications reported statistically significant positive outcomes (or at least positive trends) for survival or tumor remission and QoL, while several studies reported no effect on survival, recurrence, remission, and QoL [6]. In 2007, Kienle and Kiene published a systematic review of prospective clinical trials on anthroposophic mistletoe extracts and stated beneficial effects of VA-E application with respect to QoL and reduction of side effects of cytoreductive therapies in most analyzed trials [9]. They concluded that the best evidence for efficacy of VA-E exists for the improvement of QoL and the reduction of side effects of cytotoxic therapies, while the survival benefit was a matter of critique [9].

### 4.3. Limitations of the Used Measures of QoL and Associated Dimensions

To avoid a selection bias, we included all studies addressing QoL-associated dimensions, that is, studies using standard health-related OoL-instruments and those measuring QoL related dimensions. We will discuss putative limitations of some instruments used by the primary authors. 

The investigation of Kjaer, which used a less suited visual analogue scale (VAS) to measure well-being and QoL, was excluded from analysis anyway because the authors presented just the data of the VA-E arm [35, 36]. The investigation of Borelli [24] used the Spitzer Quality of Life Index [37] which covers relevant 5 QoL dimensions. Also the EORTC-QLQ C30 and BR 23 which incorporate 9 multi-item scales is among the best fitting QoL instruments [38]. The trial of Dold et al. [25] used a measure which refers to the Karnofsky performance status scale [39], which is the physician's rating of physical activity and self-supply, and rated additional medical conditions such as appetite, cough, dyspnea, pain, fever, and edema. These measures refer to important aspects of QoL and can be regarded as more or less suitable. However, this study relied on physician's rating rather than patient's self-assessment; nevertheless, to avoid a selection bias we decided to include this study too (in fact the treatment effects were rather low, and thus this decision would not promote very positive overall effects). The authors detected significant differences only with respect to subjective improvement of disorders—but there were no differences between the groups with respect to the outcomes. 

All other trials used a 16-item instrument to measure psychosomatic “self-regulation” [26–33, 40], which was assumed to asses an important aspect of QoL. Recently we were able to approve correlations between “self-regulation” and QoL; this unique dimension deals with competence and autonomy of patients and thus should be regarded as an active problem-solving capacity in terms of an active adaptation to stressful situations to restore well-being [41]. Nevertheless, this dimension can be regarded as QoL associated.

### 4.4. Potential Bias Factors

Potential bias factors which might contribute to the overall positive effects described in the analyses of Grossarth-Maticek et al. [26–33, 40] were discussed in detail by the authors themselves [26, 29], that is, selection bias and loose matching, Cox proportional hazard models with/without adjustments, and so forth. Because these studies started in 1973, several relevant study objectives were not available, that is, exact dates of first diagnosis, operation, initial and follow-up data assessments and matching, socio-economic status, social support, spirituality, and so forth. In these studies, attrition bias was less important because with the drop out of any study patient, the matching partner was also excluded and thus the balance of the groups was not severely affected [26, 29]. Altogether, their internal validity was limited by selection bias and confounding; moreover, there were no written protocol, no statistical hypotheses, and no sample size calculation, the sample sizes were in most cases very small. 

Another intriguing fact could be that the nonrandomized studies of Grossarth-Maticek's group might have a lower external validity (generalisability), because the inclusion and exclusion criteria were not very precise and not all of them were explicitly formulated in advance. Moreover, apart from the matching criteria, there were no explicit procedures for building pairs. The most important fact was raised by the authors themselves [26, 29, 33], as they cannot exclude the possibility that patients with a good prognosis were preferentially enrolled, since patients from both groups who died shortly after the diagnosis would not have entered the study. 

Because the investigations with a stronger internal validity yielded similar results as compared to the studies with the stronger external validity point in the same direction, one can conclude that the results are more or less consistent. Nevertheless, because several patients started with lower self-regulation score during the treatment, one cannot exclude the possibility of normalization and regression to the mean effects.

### 4.5. Explanation of Outcome Effects

**Figure 5:**
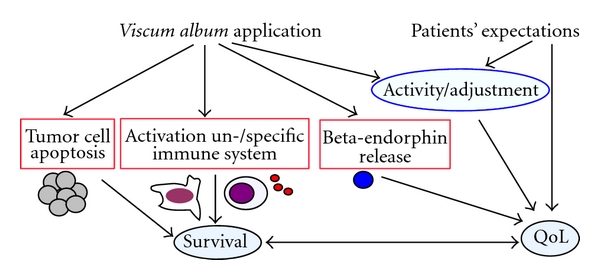
Hypothetical diagram explaining the possible mechanisms of *Viscum album* extract application on QoL.

How could the positive effects of *Iscador *on QoL-related dimensions be explained (Figure 5)? Direct pharmacological effect could depend on the applied doses, as suggested by Németh et al. [42]. Indeed, a recent study investigated different doses escalation regimes of *Iscador* and found significant differences for the QoL aspects: physical complaints, vitality, mental behavior, presence of personality, and social environment [43]; particularly the presence of personality was highest in the group with large local reactions in response to the plant extract, an effect which was significantly dependent on the dose escalation. That the plant extract by itself may be crucial for the beneficial effects is supported by findings showing that the survival of cancer patients is a function of the relative duration of *Iscador *treatment, even in patients with initially identical “self-regulation” scores [26]. But it is unclear whether the physical improvement may precede a psychical stabilization or whether a mental stabilization may have a stress-reducing effect which in turn may have a beneficial effect on the physical situation. 

One cannot ignore the fact that positive expectations of the patients are also modulating factors influencing behavior which in turn can have a strong impact on health. Expectations are also thought to underlie the so-called “placebo effects”, impacting perceptions and biological processes. In fact, invasive procedures such as injections have a higher “placebo response” compared with oral drugs [44]. One should realize that the “placebo effect” itself represents a true measurable correlate of an organism's psychoneurobiological response and, thereby, influences the healing process [45]. The “placebo effect” is taken to mean also the broad array of nonspecific effects in the patient-physician relationship, including attention, compassionate care, and the modulation of expectations; anxiety; self-awareness [46]. At least, one may suggest that all of these explanations could contribute to the observed positive effects, particularly because the application of VA-E is regarded by most patients as an active and effective chance to fight the tumor. Thus, there might be a combination of pharmacological and motivational aspects mediated by the *Iscador* application which may contribute to the positive outcome (Figure 5). Whatever the underlying effects are, a recent review of our group found that pooled analysis of clinical studies suggests that adjuvant treatment of cancer patients with the mistletoe extract *Iscador *is associated with a better survival [13], albeit we found hints for a publication bias which limits the evidence found in that meta-analysis, and this better survival is probably associated with a better QoL, too.

## 5. Conclusion

The analyzed trials give some evidence that *Iscador* treatment might have moderate beneficial short-time effects on QoL-associated dimensions and psychosomatic self regulation. We are aware that the pooled estimates are driven by quite heterogeneous data. Because the results are promising, despite of methodological limitations, large and well-designed randomized controlled trials should be funded. Subsequently, the studies may serve as models for future trials in the area.

##  Disclosure

A. Büssing and T. Ostermann received financial support with a grant from Hiscia—Verein für Krebsforschung, Arlesheim (Switzerland), which is producing the whole-plant extract. The authors were free to interpret the data according to a strict scientific rationale without any explicit demands of any company. The authors are in no way associated with any pharmaceutical company.

## Figures and Tables

**Table 1:** Overview on identified clinical studies/strata.

Ref.	Year	Number of patients		Tumor localization	Study design of all studies				QoL instrument	Outcome	
		*Iscador*	Control		control	design	matching	JADAD score		SMD	STE
[24]*	2001	20	10	Breast	PLG	Rand	—	1	Spitzer QoL Index	1.71	0.39
[25]*	1991	114	113	Lung	PLG	Rand	—	2	Karnofsky's Index + additional items	0.32	0.15
[26, 27]	2001	39	39	Multiple	PLG	Rand	MP	2	Self regulation	0.53	0.23
[26, 27]	2001	17	17	Breast	PLG	Rand	MP	2	Self regulation	0.53	0.34
[28]	2006	17	17	Breast	PLG	Rand	MP	2	Self regulation	0.67	0.34
[29]	2006	38	38	Breast	PLG	Rand	MP	2	Self regulation	0.53	0.23
[29]	2006	84	84	Breast	PLG	NR	MP	1	Self regulation	0.34	0.15
[30]	2007	41	41	Ovary	PLG	Rand	MP	2	Self regulation	1.24	0.32
[30]	2007	137	137	Ovary	PLG	NR	MP	1	Self regulation	0.45	0.16
[31]	2007	19	19	Cervix	PLG	Rand	MP	2	Self regulation	0.51	0.32
[31]	2007	102	102	Cervix	PLG	NR	MP	1	Self regulation	0.81	0.14
[32]	2007	22	22	Melanoma	PLG	Rand	MP	2	Self regulation	0.84	0.30
[33]	2008	198	198	Corpus uteri	PLG	NR	MP	0	Self regulation	0.27	0.10
[33]	2008	56	56	Corpus uteri	PLG	Rand	MP	1	Self regulation	0.62	0.19
[34]	2005	33	33	Breast	PLG	Rand	—	2	EORTC-QLQ C30, BR 23	0.59	0.25
[35, 36]	1984	14	—	Kidney	historic	—	—	—	Visual analogue Scale	—	—

PLG: parallel group; Rand: randomization; NR: no randomization; MP: matched pairs; SMD: standardized mean differences; STE: standard error; *placebo/alternative control, and thus not included in the statistical analysis.
